# A Multi-Model Strategy for Optimizing Hepatitis B Virus preS1 Epitope Recognition by the Antibody HzKR127

**DOI:** 10.3390/cimb48080791

**Published:** 2026-08-03

**Authors:** Wenqing Chen, Yuanzhong Tu, Kai Wang, Runze Xie, Pengyuan Yang, Yanan Gao, Wenxiang Huang

**Affiliations:** 1 Department of Geriatrics, The First Affiliated Hospital of Chongqing Medical University, Chongqing 400016, China; 2 Key Laboratory of Epigenetic Regulation and Intervention, Institute of Biophysics, Chinese Academy of Sciences, Beijing 100101, China; 3 University of Chinese Academy of Sciences, Beijing 100049, China

**Keywords:** hepatitis B virus, preS1, antibody engineering, computational biology, saturation mutagenesis, HzKR127

## Abstract

Functional cure of chronic hepatitis B virus (HBV) infection remains a significant challenge, making viral-entry-blocking antibodies a promising antiviral strategy. Here, we developed a structure-guided computational workflow for affinity-enhancing candidate mutations at the interface between the humanized neutralizing antibody HzKR127 and the HBV preS1 peptide epitope. Based on the crystal structure of the HzKR127–preS1 complex, we performed single-site saturation mutagenesis across the paratope, evaluating variants with a consensus effect score integrated from seven computational models. Benchmarking against published alanine scanning data showed that our consensus score effectively identified major-affinity-loss residues, achieving ROC AUC values of 0.81 and 0.84 for residue-level and site-level predictions, respectively. Mutational profiling revealed distinct asymmetric mutational responses, with broad intolerance on the preS1 side and localized favorable substitutions within antibody CDRs. Multilevel prioritization identified 26 antibody-side candidates, 17 of which showed improved HADDOCK refinement scores compared to the wild type. In particular, the H:D97W/F/Y substitutions presented the strongest structural rationale for enhancing improved interfacial packing through aromatic hydrophobic contacts with preS1 Phe10. These findings provide a prioritized list of candidates for experimental validation and a practical framework for the rational optimization of antibodies targeting functionally constrained viral epitopes.

## 1. Introduction

Hepatitis B virus (HBV) infection remains a major cause of chronic liver disease, cirrhosis and hepatocellular carcinoma [1,2]. Although current antiviral therapies efficiently suppress viral replication, functional cure is still achieved in only a limited proportion of patients. Strategies that block viral entry and facilitate the development of new antiviral molecules therefore remain of substantial interest [3,4]. The preS1 region of the large HBV surface protein contributes to viral attachment and entry through engagement of the hepatocyte receptor sodium taurocholate cotransporting polypeptide (NTCP), which also serves as a receptor for hepatitis D virus. This region contains experimentally defined neutralizing epitopes and is therefore an attractive target for entry-blocking antibodies [5,6,7].

HzKR127 is a humanized derivative of the broadly neutralizing anti-HBV antibody KR127 which recognizes an epitope within the preS1 region. The crystal structure of the HzKR127 Fab bound to the preS1 peptide has been identified (Protein Data Bank ID: 2EH8) [8]. Using this structural framework, Chi et al. performed experimental alanine scanning and identified key interface residues, particularly within heavy-chain complementarity-determining region 3 (HCDR3), that contribute to antigen recognition [8]. Subsequent structure-guided mutagenesis and affinity maturation studies further improved the binding capacity of selected CDR residues [9]. These observations indicate that, despite its established neutralizing activity, the HzKR127 interface may retain exploitable mutational space for further optimization.

Previous optimization efforts have largely depended on empirical site-directed mutagenesis and have not comprehensively interrogated the full substitution landscape of the interface. Alanine scanning is valuable for defining binding hot spots, but it mainly reports the disruptive consequence of side-chain truncation and does not determine whether alternative side-chain chemistries may be beneficial [10]. Computational saturation mutagenesis can address this limitation; however, individual prediction methods differ in energy functions, parameterization and structural assumptions. Predictions from a single model may therefore be biased by method-specific scoring behavior [11,12,13,14,15,16,17].

For antibody–peptide interfaces, key limitations apply: energy-based methods (FoldX, PyRosetta) do not fully capture mutation-induced CDR backbone flexibility, while empirical models (mCSM-PPI2, SSIPe) are constrained by template bias or shallow viral evolutionary profiles. Because several methods share related structural assumptions, consensus integration was utilized to improve relative ranking robustness rather than estimate absolute binding free energies. Structurally, this candidate-prioritization workflow differs from whole-chain sequence and scaffold de novo generators—including IgFold [18], PROT-ON [19], and AbDesign [20]—which focus on broader de novo design rather than refining established native interfaces.

In contrast, the present workflow focuses on interpretable local mutation prioritization around an experimentally determined antibody–peptide complex. A robust antibody-engineering workflow should integrate mutation-effect magnitude, cross-model agreement, literature-derived experimental annotation and structural interpretability [21].

Here, we used the HzKR127-preS1 complex as a model system to establish a computational screening workflow that combines interface-constrained saturation mutagenesis, multi-model consensus scoring and structural reassessment. We aimed to define the mutational designability of the antigen–antibody interface and to prioritize antibody-side substitutions for subsequent experimental validation. This work provides candidate mutations for the rational optimization of HzKR127 and illustrates a computational prioritization workflow developed for the HzKR127–preS1 system that may inform future antibody-engineering studies after validation in additional antibody–antigen complexes.

## 2. Materials and Methods

### 2.1. Structural Template and Saturation Mutagenesis

The HzKR127-preS1 antigen–antibody complex (PDB ID: 2EH8; X-ray diffraction; resolution, 2.60 Å) was used as the structural template [8]. The antibody heavy chain, antibody light chain and preS1 epitope peptide were uniformly assigned as chains H, L and P, respectively. The analyzed region included the heavy- and light-chain CDRs and the resolved preS1 epitope peptide. Each included position was subjected to single-site saturation mutagenesis by replacing the wild-type residue with each of the other 19 standard amino acids. Wild-type self-substitutions were excluded from downstream analyses as they do not represent actual mutation events.

### 2.2. Multi-Model Scoring and Consensus Effect Score

Mutation effects were predicted using seven models: FoldX 5.1, HADDOCK Basic (HADDOCK3), HADDOCK Alascan (HADDOCK3), PyRosetta-4 (release 2026.03), PROT-ON v1.1/EvoEF1, the mCSM-PPI2 web server and the SSIPe web server [11,12,13,14,15,16,17]. These models were selected to capture complementary structure-based, energy-based and sequence/evolution-informed mutation-effect estimates, rather than to provide a single definitive prediction of binding affinity. Each model was executed according to the recommended public server or local deployment workflow. Apart from necessary structure preprocessing, mutation construction and local optimization, default parameters were retained. FoldX calculations were performed in triplicate and averaged. PyRosetta mutant structures were locally optimized and scored with the ref2015 energy function. HADDOCK-derived scores were obtained using standard topology rebuilding, energy minimization or alanine scanning procedures.

Because raw model outputs differed in score orientation and numerical scale, all scores were first directionally harmonized so that larger values indicated more favorable predicted effects on antigen–antibody binding. To reduce the influence of model-specific outliers, we then applied a robust two-sided hyperbolic tangent normalization rather than Z-score normalization, min–max scaling or quantile normalization [22]. For each model, the 76th percentile of the positive score distribution and the 76th percentile of the absolute negative score distribution were calculated separately and denoted as *c_pos_* and *c_neg_*, respectively. For a direction-adjusted score *x*, the normalized score *s* was defined as

s=tanh(xcpos), x>0−tanh(|x|cneg), x<00, x=0

The 76th percentile was used as an empirical scaling point because tanh (1) = 0.7616; thus, scores reaching the corresponding 76th percentile were mapped to a normalized magnitude of approximately 0.76. This transformation preserved continuous variation among moderate signals while progressively compressing stronger signals toward saturation, thereby limiting the contribution of extreme values. Separate scaling of positive and negative scores further accounted for asymmetry between favorable and deleterious score distributions within individual models. The normalized scores were constrained to [−1, 1], with positive values indicating predicted favorable substitutions and negative values indicating predicted disruptive substitutions. For each mutation, the median of the seven normalized model scores was defined as the consensus effect score. Model-support count was defined as the number of models assigning a positive normalized score to a given substitution, with model disagreement quantified as the standard deviation of the seven normalized scores. For all analyzed substitutions, all seven models returned valid numerical outputs; zero-valued outputs were retained as neutral predictions rather than missing values. At the site level, the median supporting model count was calculated across the 19 non-wild-type substitutions at each residue to summarize the typical level of cross-model support.

An integrated priority score was also calculated to support candidate ranking. For each model, direction-adjusted scores were ranked in descending order and converted into relative priority values. Relative ranks across the seven models were then aggregated to capture ranking stability in addition to absolute consensus effect size.

### 2.3. Literature-Based Validation and Candidate Selection

Published data for the HzKR127-preS1 interface were collected for external validation and literature-based residue annotation [8,9]. The ratio between mutant and wild-type dissociation constants (*K_d_MUT*/*K_d_WT*) was extracted when available. Mutations with *K_d_MUT*/*K_d_WT* > 10 were defined as major-affinity-loss mutations, whereas mutations with 0 < *K_d_MUT*/*K_d_WT* ≤ 10, including limited loss, neutral change and reported affinity improvement, were grouped as limited-affinity-loss mutations. The receiver operating characteristic (ROC) analysis was conducted directly on the native binary classification of major-affinity-loss versus limited-affinity-loss mutations (with major-affinity-loss treated as the positive class) as defined in our published reference datasets [8].

Receiver operating characteristic (ROC) analysis was performed using both alanine substitution scores and site-median scores. The site-median score was calculated as the median consensus effect score across the 19 non-wild-type substitutions at a given residue and was used to describe overall positional mutational sensitivity. For the site-level validation analysis, site-median scores were evaluated for the 49 antibody CDR residue sites with available published alanine scanning data. These sites were assigned to the major-affinity-loss or limited-affinity-loss group according to the published experimental data. Lower site-median scores indicate greater predicted vulnerability to substitution at the corresponding residue.

To approximate the limited throughput of experimental validation, top-k enrichment analysis was performed at k = 3, 5 and 10 to evaluate whether literature-defined hot and warm spots were preferentially recovered among the top-ranked alanine-sensitive positions. Interface residue sites were ranked according to the alanine substitution consensus effect score from low to high. The observed cumulative recovery was compared with the theoretical optimal recovery and the random-ranking expectation.

Candidate selection incorporated the consensus effect score, model-support count and ranking stability. Site-level designability was defined as the proportion of non-wild-type substitutions at a position with a consensus effect score greater than zero. The best non-alanine substitution score was used to approximate the potential upper bound of improvement at each position. Priority was assigned to antibody CDR mutations with high consensus scores, strong multi-model support and a plausible structural basis for improvement.

### 2.4. Structural Refinement and Interaction Analysis

Wild-type and mutant complexes were refined from the bound coordinates (PDB 2EH8) using HADDOCK3 topoaa–flexref–mdref–emref refinement workflow [12]. Flexref (sampling factor = 10) generated 10 semi-flexible models, each refined across 10 explicit-solvent runs (100 final structures; mdref: 200 minimization, 100 heating, 1250 MD, and 500 cooling steps; emref: 200 minimization steps). Six Cα–Cα distance restraints (±2.0 Å tolerance) were applied: two intra-antibody (A193–A350 and A134–A236) and four wild-type interface anchors (L91–P5, L32–P9, H35–P7, and L27D–P3). The structure with the lowest HADDOCK score was selected for comparison.

To establish a random baseline, 156 control mutations (six sets of 26 non-wild-type single-point substitutions, excluding the 26 prioritized candidates) were selected from the same CDR mutagenesis space and evaluated using the identical HADDOCK3 refinement protocol, distance restraints, and scoring thresholds.

All complexes were mapped back to a three-chain representation consisting of the heavy chain, light chain and preS1 peptide. Structures were aligned using the antibody heavy- and light-chain backbones as references. HADDOCK score, van der Waals energy, electrostatic energy, desolvation energy, buried surface area and changes in local hydrogen bonds, salt bridges, hydrophobic contacts and aromatic interactions were compared between wild-type and mutant complexes. Structural visualizations and distance measurements were performed using PyMOL (v3.2.0a; Schrödinger, LLC, New York, NY, USA).

### 2.5. Statistical Analysis

Skewed continuous variables are reported as medians with interquartile ranges. Categorical variables are reported as counts or percentages. Differences between two groups of continuous variables, including consensus effect scores and HADDOCK scores, were assessed using a two-sided Mann–Whitney U test. For the random-control analysis, the proportions of mutations with HADDOCK scores more favorable than the wild-type complex were compared using an odds ratio estimated by the Woolf method, with the corresponding 95% confidence interval and two-sided (*p*) value. Classification performance was evaluated using ROC curves and the area under the curve (AUC). Ranking performance was evaluated by top-k enrichment analysis. A two-sided *p* value < 0.05 was considered statistically significant.

## 3. Results

### 3.1. Multi-Model Consensus Scores Are Consistent with Experimental Alanine Scanning Data

**Figure 1:**
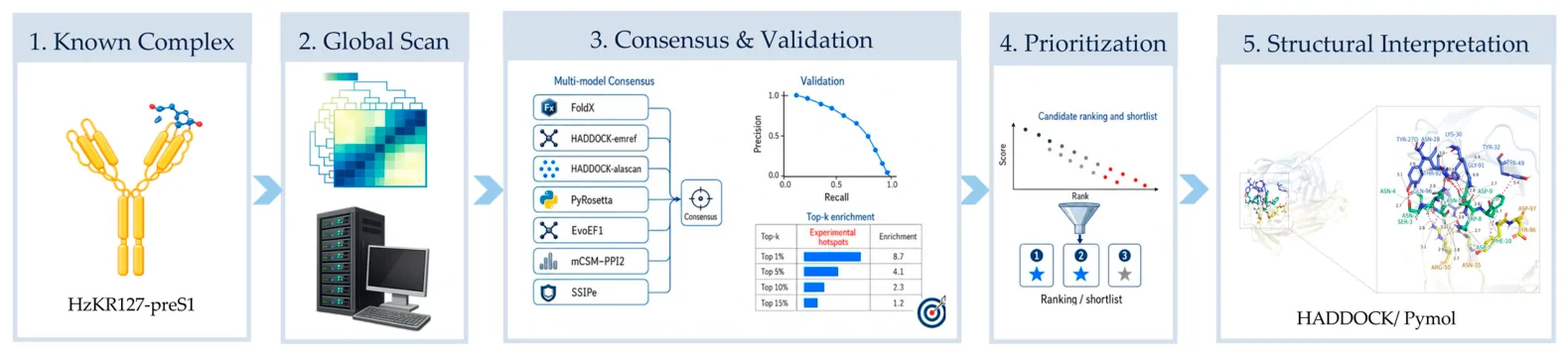
Computational workflow for multi-model saturation mutagenesis and candidate prioritization of the HzKR127–preS1 interface. The workflow included structure preparation of the HzKR127–preS1 complex, interface-constrained single-site saturation mutagenesis, multi-model scoring, score normalization and consensus integration, validation against published alanine scanning data, candidate prioritization and structural interpretation by HADDOCK refinement.

To mitigate the scoring bias inherent to individual models, seven mutation-effect prediction methods were integrated into a multi-model consensus workflow (Figure 1). After direction harmonization and normalization, the median normalized score across models was designated as the consensus effect score. Lower values indicated a higher probability that a given mutation would destabilize HzKR127-preS1 binding.

Published experimental mutation data for the HzKR127-preS1 interface were used to validate the scoring scheme and to annotate interface residues. The literature-derived residue annotations included hot spots, warm spots, affinity-enhancing sites, null spots and other residues. These annotations were used as external reference labels and were not generated by the seven computational models.

**Figure 2:**
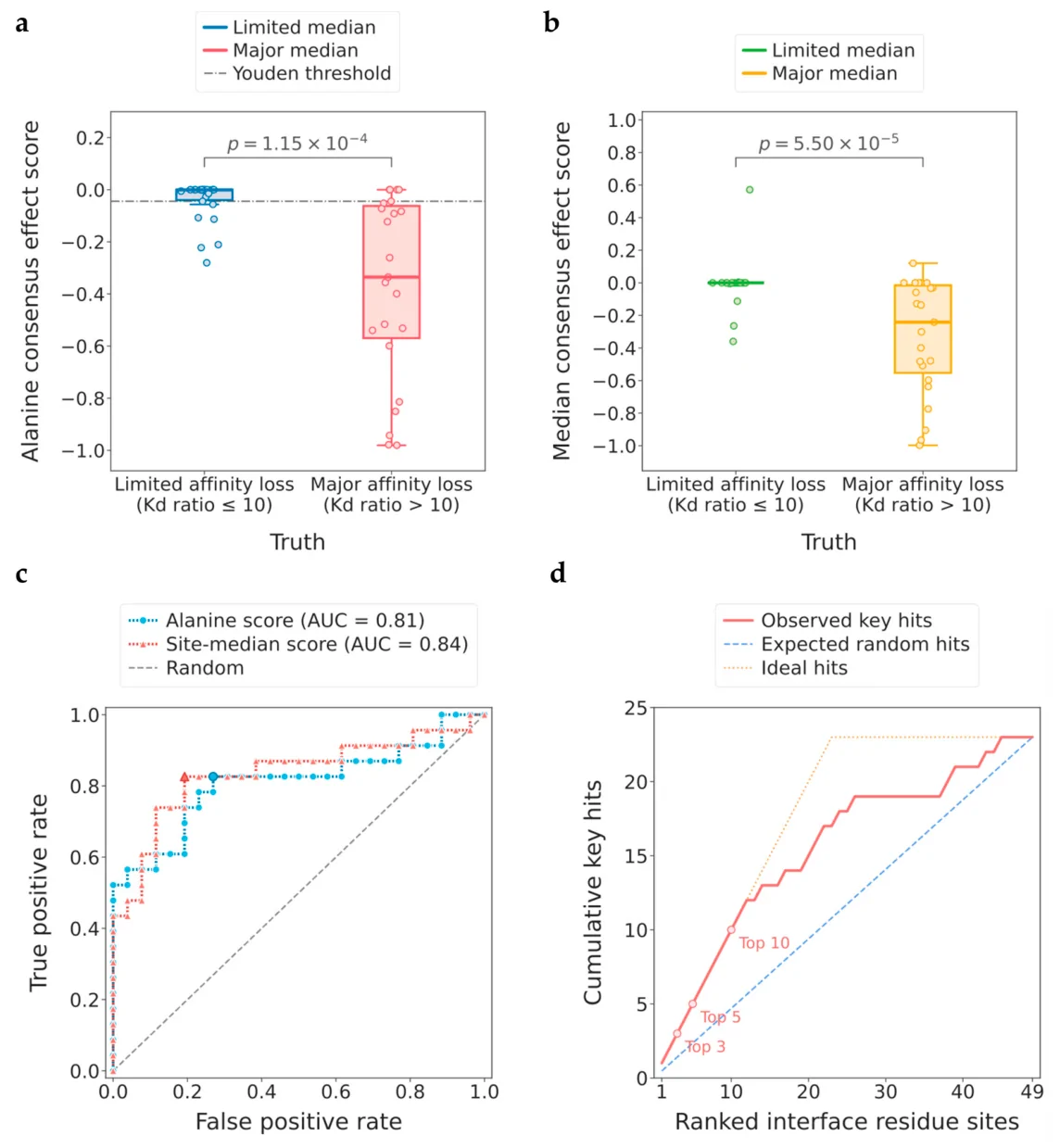
Validation of the multi-model consensus scoring strategy against published experimental mutation data. (**a**) Alanine substitution consensus effect scores in limited-affinity-loss and major-affinity-loss mutations. Each point represents one alanine mutant from the published alanine scanning dataset. Mutations with *K_d_MUT*/*K_d_WT* > 10 were classified as major-affinity-loss mutations, whereas mutations with *K_d_MUT*/*K_d_WT* ≤ 10, including limited loss, neutral effects, or reported affinity improvement, were classified as limited-affinity-loss mutations. (**b**) Comparison of site-median consensus effect scores between major-affinity-loss and limited-affinity-loss groups among the 49 antibody CDR residue sites with published alanine-scanning data. The site-median score is the median of the seven-model consensus scores across all 19 non-wild-type substitutions at each residue. Group assignments are based on published alanine-scanning data, with lower site-median scores indicating greater predicted mutational vulnerability. (**c**) ROC analysis for identifying major-affinity-loss residues (with major-affinity-loss as the positive class) using alanine-substitution scores and site-median scores. (**d**) Top-k enrichment analysis showing the cumulative recovery of defined hot and warm spots among interface residue sites ranked by alanine-substitution consensus effect score from low to high. The observed recovery was compared with the theoretical optimal recovery and the random-ranking expectation. Statistical significance was assessed using the Mann–Whitney U test. ROC—receiver operating characteristic; AUC—area under the curve.

Alanine consensus scores were significantly lower in the major-affinity-loss group than in the limited-loss group, indicating that the computational alanine substitution consensus score was consistent with experimentally observed disruptive effects (Figure 2a; each point represents one alanine mutant; Mann–Whitney U test, *p* = 1.15 × 10^−4^). When the median score across all 19 non-wild-type substitutions was used to represent overall site sensitivity, residue sites in the major-affinity-loss group showed significantly lower site-median consensus scores than those in the limited-affinity-loss group (Figure 2b; each point represents one residue, *p* = 5.50 × 10^−5^). This result indicates that residue sites showing marked experimental sensitivity to alanine replacement also tended to exhibit broader predicted vulnerability across the computational substitution profile.

ROC analysis showed that both alanine substitution scores and site-median scores identified literature-annotated binding-contribution residues, with AUC values of 0.81 and 0.84, respectively (Figure 2c). Top-k enrichment analysis further showed that literature-defined and warm spots were preferentially recovered among top-ranked alanine sensitive positions, exceeding the random-ranking expectation (Figure 2d). In this analysis, residue sites were ranked by alanine substitution consensus effect score from low to high. The top 10 sites were L32, L91, L27D, H96, H50, L94, L96, H33, H35 and L34. The ranked site information is provided in Supplementary Table S1. The performance of the consensus score is further illustrated in Supplementary Figure S1, showing the confusion matrix at the optimized threshold and the precision–recall curve for experimentally defined major-affinity-loss sites.

**Figure 3:**
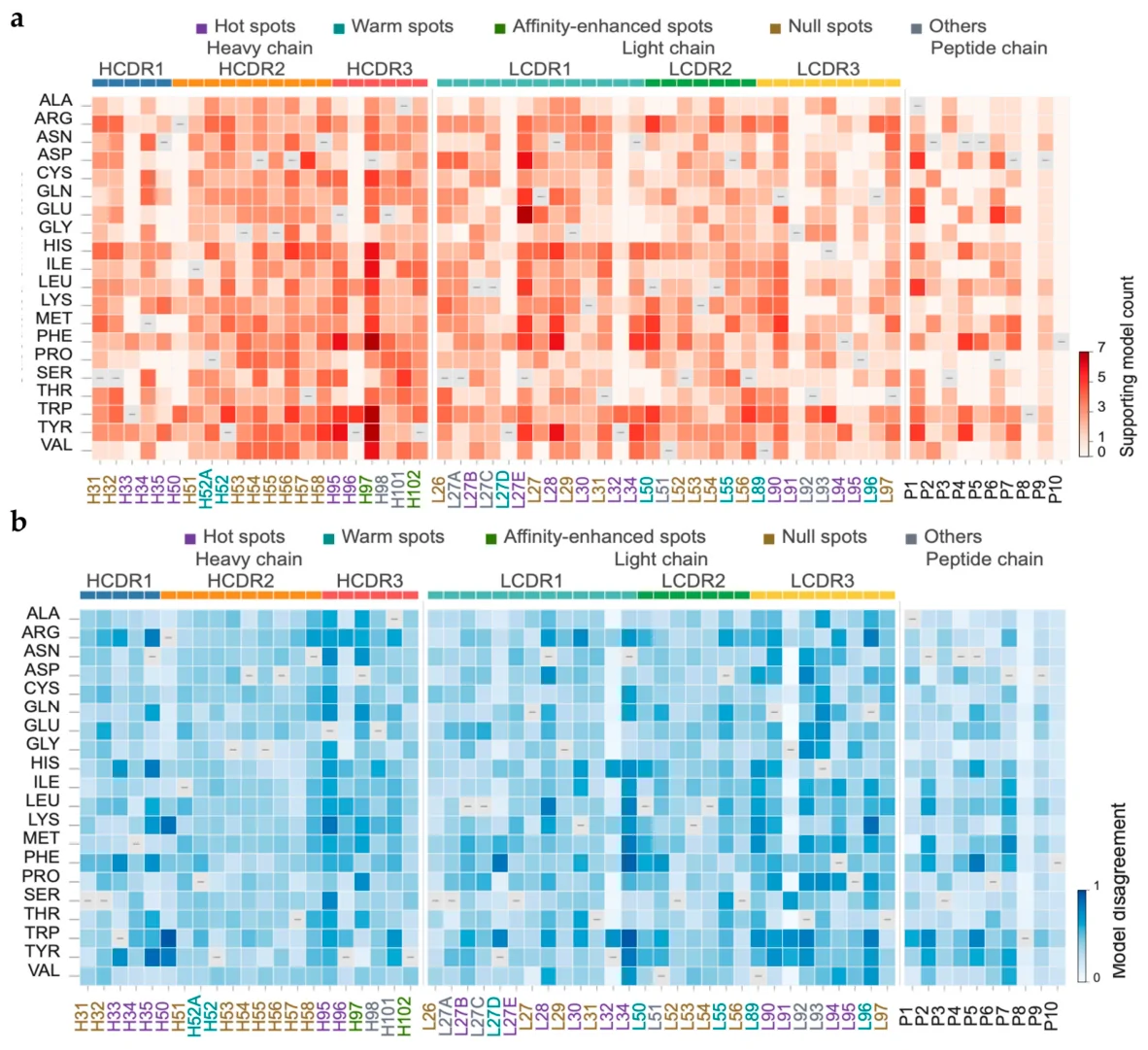
Saturation-mutagenesis landscape and model-agreement profile of the HzKR127–preS1 interface. (**a**) Heatmap of supporting model counts for single-site saturation mutations across antibody CDR residues and resolved preS1 peptide residues. The supporting model count indicates the number of models, among the seven computational models, that predicted a given substitution to be favorable. (**b**) Heatmap of model disagreement for the same mutation matrix. Model disagreement was calculated as the standard deviation of normalized scores across the seven models. Residue annotations indicate literature-defined hot spots, warm spots, affinity-enhanced spots, null spots and other residues. HCDR—heavy-chain complementarity-determining region; LCDR—light-chain complementarity-determining region.

The interface mutation matrix exhibited heterogeneous patterns of cross-model support and inter-model disagreement, with some mutation-effect signals supported by multiple models and a subset of mutations showing substantial prediction differences among models (Figure 3a,b). Together, these data support the use of the multi-model consensus score and model agreement for downstream screening of antibody CDR mutations.

### 3.2. The HzKR127–preS1 Interface Exhibits Antigen-Side Constraint and Antibody-Side Mutational Plasticity

After validating the consensus scoring framework, we compared the mutational responses of the two sides of the complex: the antibody CDRs and the preS1 epitope. This analysis was designed to define interfacial rigidity and plasticity and to establish a rational search space for antibody engineering.

**Figure 4:**
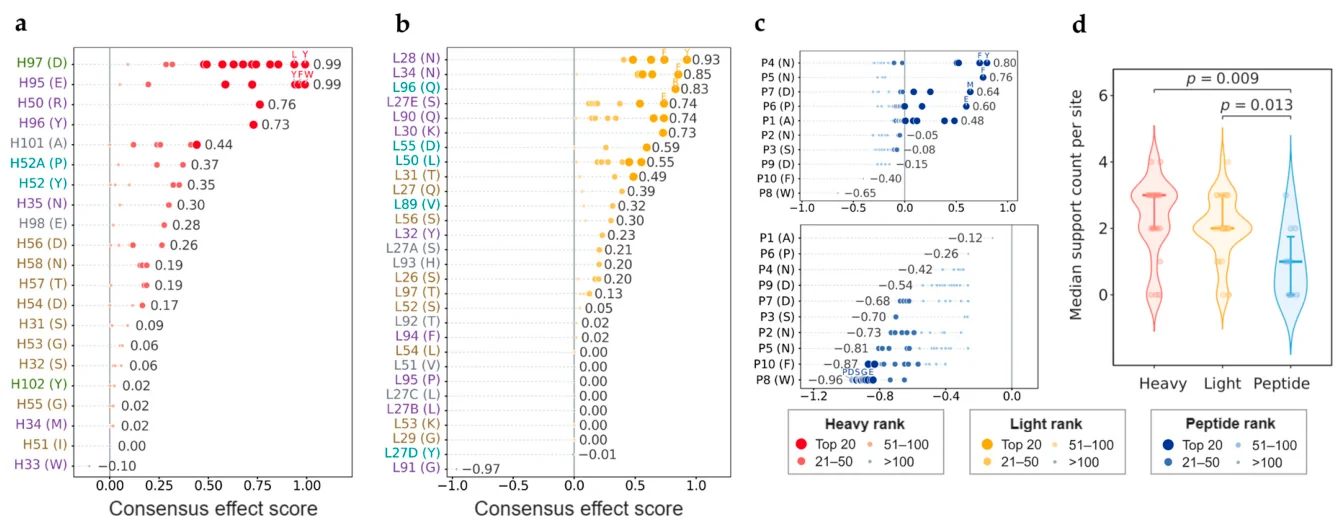
Chain-specific distribution of predicted favorable substitutions at the HzKR127–preS1 interface. (**a**) Heavy-chain CDR residues. (**b**) Light-chain CDR residues. (**c**) preS1 peptide residues. Each dot represents one specific non-wild-type single amino acid substitution. The x-axis represents the consensus effect score, and the dashed vertical line indicates zero. Positive scores indicate predicted favorable substitutions, whereas negative scores indicate predicted deleterious substitutions. Top 100 substitutions are plotted per chain. Varying dot numbers across residues reflect this visualization rule; all 19 substitutions were evaluated at all positions. (**d**) Comparison of site-level median supporting model counts among heavy-chain CDR, light-chain CDR, and preS1 residues. Each point represents one residue site, and violin plots show the distribution. The supporting model count is the number of models (out of seven) assigning a positive normalized score to a substitution. The site-level median, calculated across the 19 non-wild-type substitutions at each residue, reflects typical cross-model support rather than the number of favorable substitutions. Pairwise statistical comparisons are shown above.

The global distribution of candidate substitutions revealed pronounced asymmetry across the interface (Figure 4a–c). Each dot represents one specific non-wild-type substitution. We displayed the top 100 substitutions per chain ranked by consensus score, also including the highest-scoring mutant for residues with no mutations in the top 100. The dot density reflects this rank-based threshold rather than unequal evaluation; all 19 substitutions were evaluated at all residues. Heavy- and light-chain CDRs contained numerous predicted favorable substitutions, and several positions accumulated multiple high-scoring candidates. In contrast, the preS1 epitope showed only sparse and weak positive signals, whereas most substitutions were predicted to be deleterious. Model-support analysis further confirmed that high-scoring antibody-side candidates had stronger cross-model support, whereas the preS1 side lacked stable and reproducible gain-of-function signals (Figure 4d).

The broad intolerance of preS1 substitutions observed in our analysis reflects their predicted effects on HzKR127 recognition. Although the preS1 region participates in NTCP-mediated HBV entry, the present computational study evaluates antibody-binding effects rather than viral entry, NTCP engagement, or viral fitness. Therefore, the antigen-side mutational pattern should be interpreted strictly as a predicted antibody-recognition constraint.

These observations indicate that the HzKR127-preS1 complex is not equally optimizable from both sides of the interface. The antigenic epitope offers limited exploitable sequence space because of structural and functional constraints, whereas the antibody CDRs retain localized conformational and chemical plasticity. Subsequent affinity-maturation candidate design was therefore focused on antibody CDR residues.

### 3.3. Favorable CDR Mutations Are Locally Clustered and Exhibit Physicochemical Preferences

**Figure 5:**
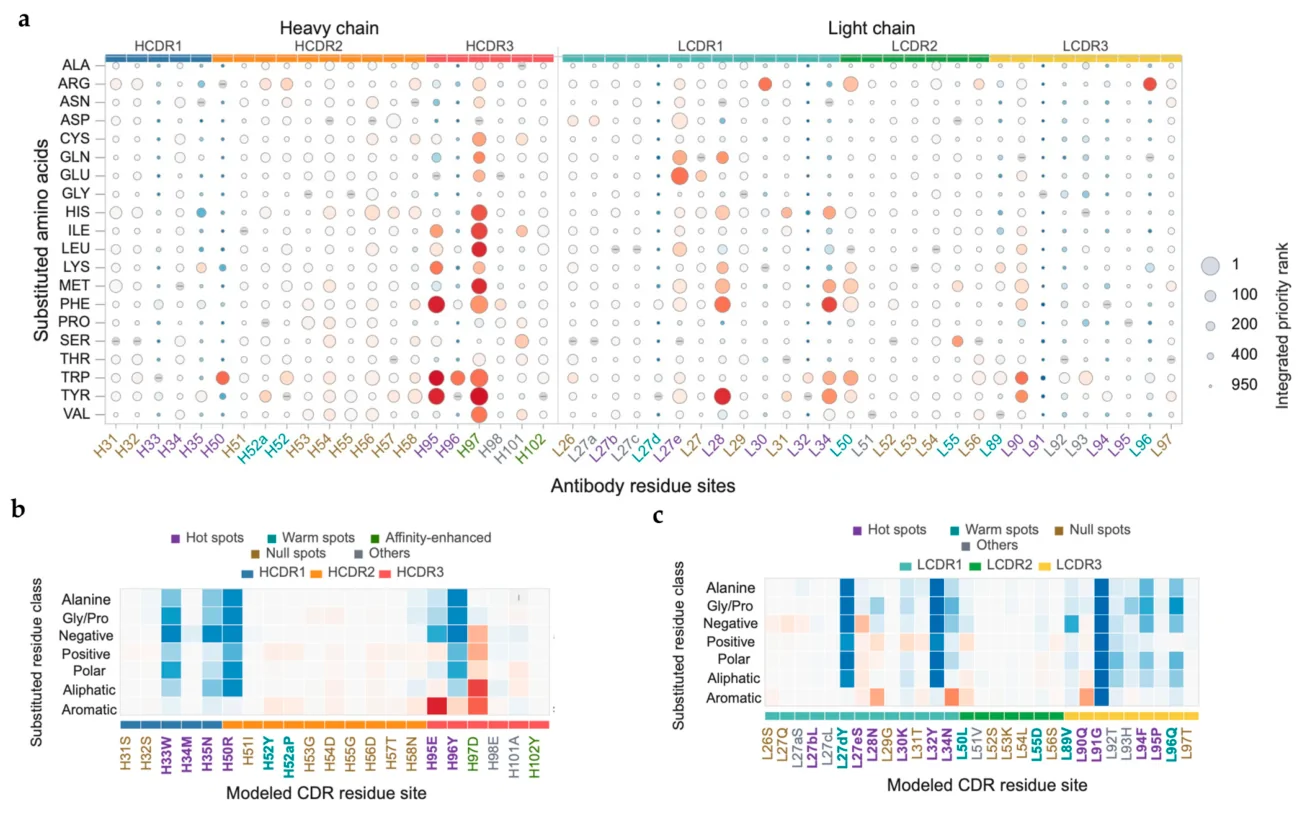
Localized designable regions and physicochemical preferences within antibody CDRs. (**a**) Bubble plot of candidate substitutions across antibody CDR residues. Dot color indicates the consensus effect score(ranging from −1 to 1), and dot size indicates the integrated priority rank. The text colors of residue labels on the x-axis indicate literature-derived functional annotations: red for hot spots, orange for warm spots, green for affinity-enhanced sites, blue for null spots, and gray for other residues. (**b**) Physicochemical-class preference map for heavy-chain CDR residues. (**c**) Physicochemical-class preference map for light-chain CDR residues. In panels (**b**,**c**), substituted amino acids were grouped by physicochemical class, and the color scale represents the mean consensus effect score for each class at each residue site.

To localize the source of antibody-side designability, we next examined mutation effects across CDR positions and substitution classes. Top-ranked favorable substitutions were not uniformly distributed across all CDR residues. Instead, they clustered in discrete local regions, including H95, H97, L28, L34, L96 and H50 (Figure 5a). Thus, antibody-side designability reflects a set of local interfacial microenvironments rather than broad mutational tolerance across the entire binding surface.

Substitution-class analysis showed that distinct CDR regions favored different physicochemical groups (Figure 5b,c). Around H95, H96 and H97, favorable substitutions were enriched for aromatic or bulky hydrophobic residues, such as tyrosine and tryptophan, suggesting that improved aromatic and hydrophobic packing may enhance local interface complementarity. Some light-chain positions were more responsive to polar or charged substitutions, implying that hydrogen bond networks or electrostatic complementarity may contribute to optimization in these regions.

Accordingly, the prioritized CDR candidates were not defined by score ranking alone. They exhibited structured selection patterns shaped by local geometry and chemical environment [20]. These findings provided a mechanistic basis for subsequent candidate prioritization and structural interpretation.

### 3.4. Multilevel Filtering Converges on Antibody CDR Candidate Mutations

**Figure 6:**
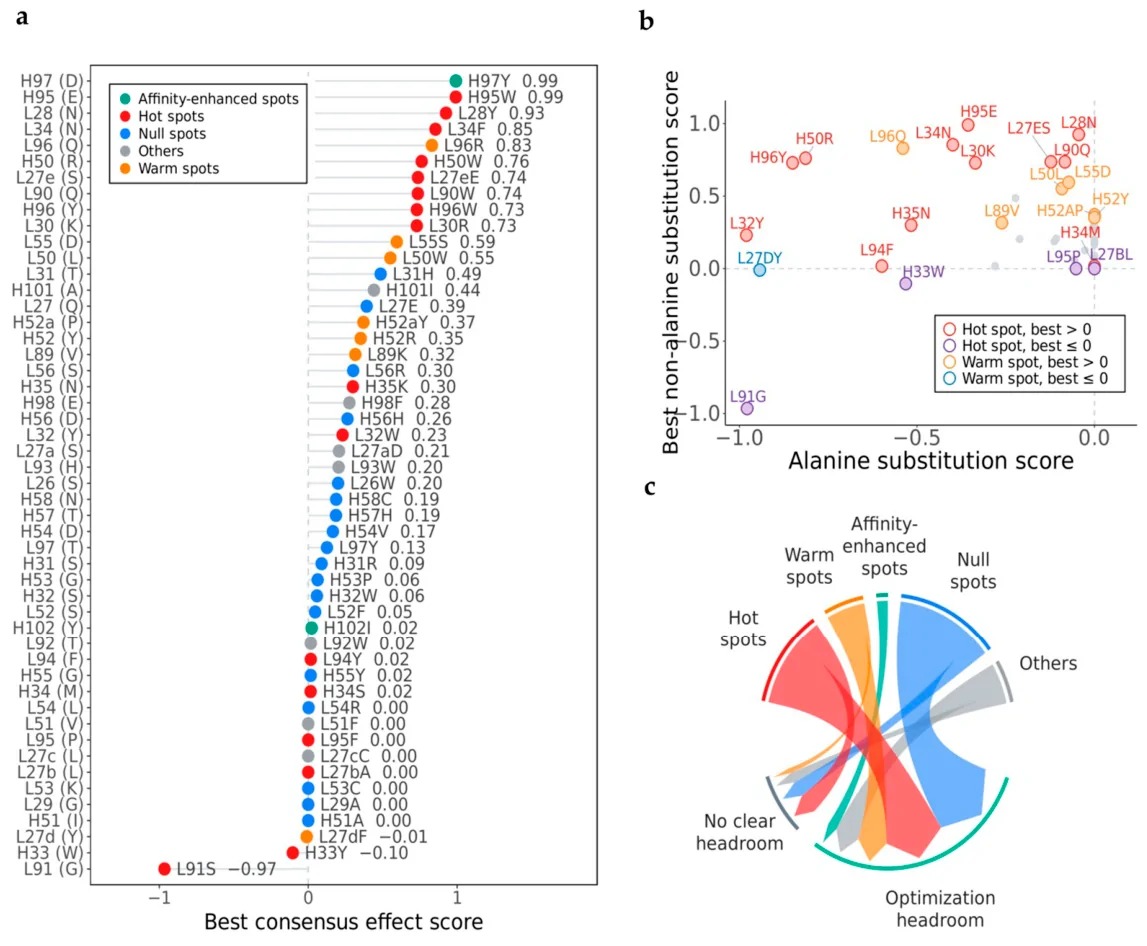
Residue-level comparison between native binding contribution and predicted engineering potential. (**a**) Ranking of antibody CDR residue sites according to the best non-alanine consensus effect score (the highest seven-model consensus score among non-alanine substitutions at each residue). Higher values indicate greater predicted potential for favorable binding effects. Dot colors indicate literature-derived residue annotations: red for hot spots, orange for warm spots, green for affinity-enhanced sites, blue for null spots, and gray for other residues. (**b**) Two-dimensional comparison of alanine substitution scores and best non-alanine consensus effect scores. Alanine substitution scores reflect the predicted effect of side-chain truncation, whereas best non-alanine substitution scores represent the highest predicted gain from alternative side-chain replacement. (**c**) Flow relationship between literature-defined functional categories and predicted optimization outcomes. Categories in panel (**c**) are color-coded to match the annotations in (**a**,**b**); ‘No clear headroom’ indicates sites where no non-alanine substitutions were predicted to be more favorable than the wild-type.

Having established the antibody CDRs as the principal designable region, we integrated literature-based functional annotation, non-alanine gain potential and multi-model consistency to refine the candidate set. Among the top 12 antibody CDR residue sites ranked by the best non-alanine consensus effect score, 11 were located at literature-defined functional hot spots; among the top 20 sites, 15 were derived from hot-spot residues (Figure 6a). The best non-alanine consensus score represents the maximum seven-model score among non-alanine substitutions at each residue, where higher values indicate greater predicted affinity-enhancement potential. This enrichment indicates that high-scoring candidates preferentially occur at residues known to participate in binding rather than at random interface positions.

Functional contribution, however, is not synonymous with engineering potential. Alanine-substitution scores primarily capture the effect of side-chain truncation on the existing binding mode, whereas the best non-alanine consensus effect score reflects the predicted optimization potential under alternative side-chain chemistry. The weak correlation between these two measurements (Figure 6b; R^2^ = 0.074) indicates that the optimal substitution at a site cannot be inferred from its alanine-scan effect alone. Destructive-effect assessment and gain-of-function screening should therefore be treated as related but distinct analytical tasks. Literature-defined hot spots, warm spots and low-contribution sites also did not correspond to a single engineering outcome (Figure 6c), arguing against the assumption that every experimental hot spot is an optimal engineering target.

**Figure 7:**
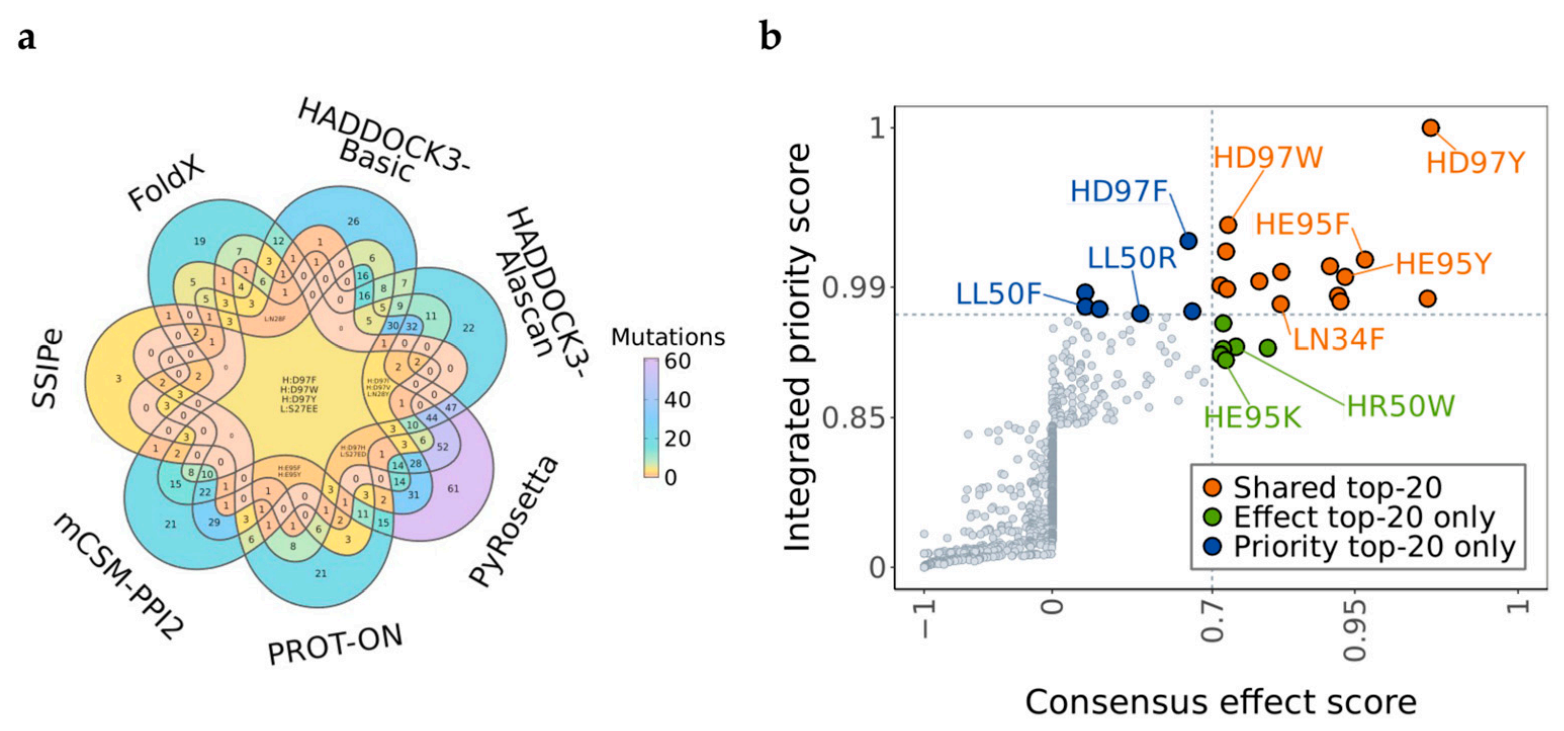
Multi-model prioritization of antibody CDR candidate mutations. (**a**) Overlap of candidate mutations selected by different computational models and ranking strategies. (**b**) Two-dimensional prioritization based on consensus effect score and integrated priority score. Orange points indicate mutations shared by the top-20 lists from both ranking strategies, green points indicate mutations selected only by the consensus effect score, and blue points indicate mutations selected only by the integrated priority score.

To improve ranking robustness, model agreement and the integrated priority score were incorporated. Model consistency analysis further showed that the integrated priority ranking maintained agreement with individual model rankings across different top-k cutoffs (Figure S2). Several candidates were supported by six or seven of the seven models. The union of the top 20 candidates defined by consensus effect score and integrated priority score contained 26 antibody CDR mutations (Figure 7a,b). These mutations combined predicted gain, functional relevance and model stability and were therefore advanced to HADDOCK structural reassessment.

### 3.5. HADDOCK Refinement Suggests Local Interface-Gain Mechanisms for High-Priority Candidates

**Figure 8:**
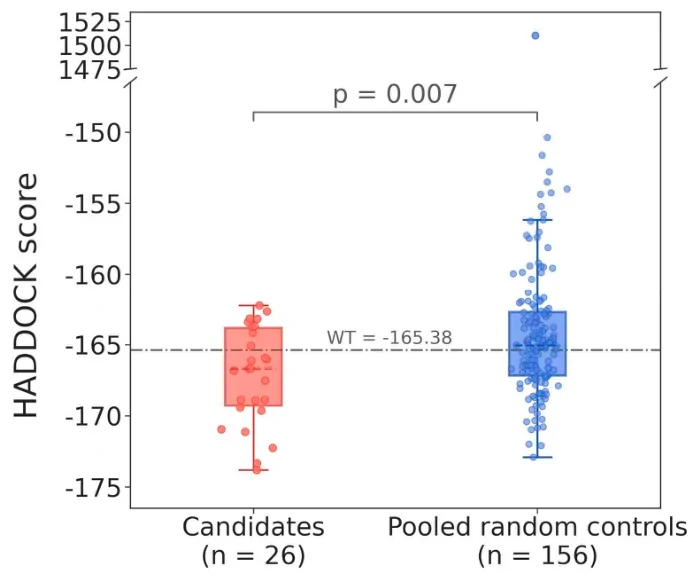
HADDOCK score distributions of prioritized candidates and random CDR controls. All variants were refined using the same SF10 workflow. Dashed horizontal line indicates the wild-type baseline (kcal/mol; lower scores represent more favorable binding energy). Statistical significance was assessed by a two-sided Mann–Whitney U test. The y-axis incorporates a break near the top to accommodate a single high-energy outlier while preserving distribution resolution near the baseline. Boxplot colors distinguish candidate mutations from random controls as labeled in the figure.

The 26 filtered candidates were subjected to HADDOCK3 refinement and ranked using the SF10 native interface HADDOCK score. Prioritized candidates achieved a significantly higher rate of HADDOCK score improvement than random controls (65.4% [17/26] vs. 42.9% [67/156]; OR = 2.51, 95% CI: 1.05–5.98, two-sided *p* = 0.0378, Woolf’s). Comparison of full score distributions confirmed significantly lower (more favorable) refined complex energies in the prioritized cohort (two-sided *p* = 0.0071, Mann–Whitney; Figure 8). These findings demonstrate robust in silico enrichment of structurally plausible substitutions, representing computational prioritization hypotheses rather than experimentally validated affinity increases (per-mutation data in Table S2).

The top-ranked mutants exhibited several types of local interfacial change, including aromatic or hydrophobic compensation, local polar reconnection, expansion of light-chain-side contacts and broader interface rearrangement (Table 1). These patterns suggest that HzKR127 optimization may depend on remodeling specific CDR microenvironments rather than on a single universal mechanism. Because HADDOCK scores are not calibrated measures of experimental binding free energy in this system, these results should be interpreted as structural plausibility assessments for candidate prioritization.

**Figure 9:**
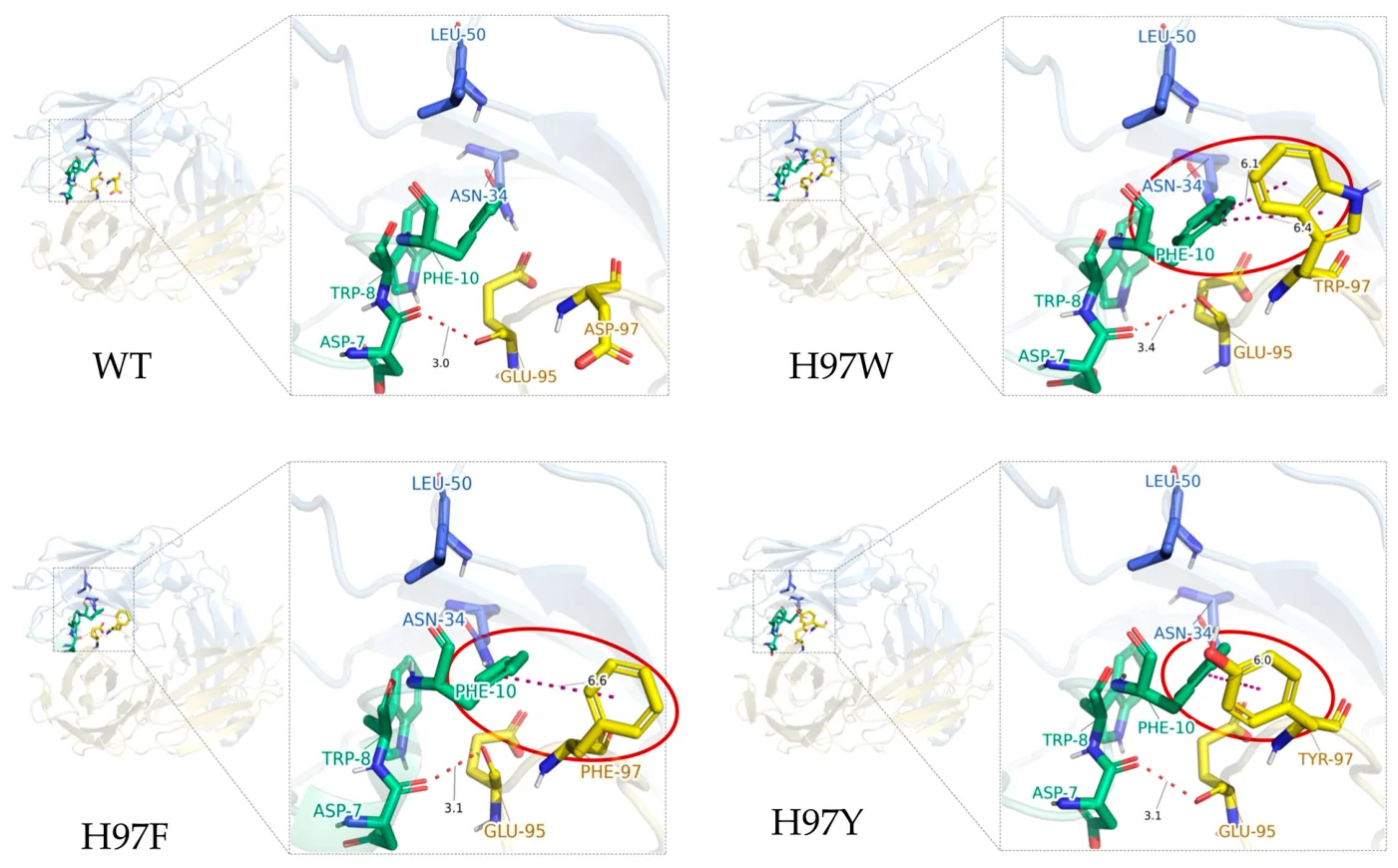
HADDOCK-refined structural comparison of H97 candidate mutations. Local structural views of the wild-type complex and H97 mutants, including H97W, H97F and H97Y, are shown. Each panel contains an overall view of the HzKR127–preS1 complex and an enlarged view of the local interface around residue H97. The red ellipses indicate the main regions of local contact changes after mutation. The heavy chain, light chain and preS1 peptide are shown in yellow, blue and green, respectively. Aromatic substitutions at H97 may alter local packing around the C-terminal region of the preS1 peptide, particularly near Phe10.

Residue H:D97 is located in the HCDR3 contact region, where HCDR3 conformation and side-chain orientation often exert major effects on antigen recognition. Among the high-ranking candidates, H:D97 substitutions showed the most coherent structural rationale (Figure 9). H:D97W, H: D97F and H:D97Y are aromatic substitutions and may reinforce aromatic or hydrophobic contacts between H97 and the region surrounding P: Phe10 of the preS1 peptide. These substitutions may thereby improve shape complementarity and interfacial packing between HCDR3 and the C-terminal aromatic region of the preS1 peptide. H:D97W/F/Y are therefore structurally interpretable candidates for priority experimental validation. However, these interpretations are based on static refined structures and should be considered mechanistic hypotheses rather than direct evidence of improved binding affinity.

**Figure 10:**
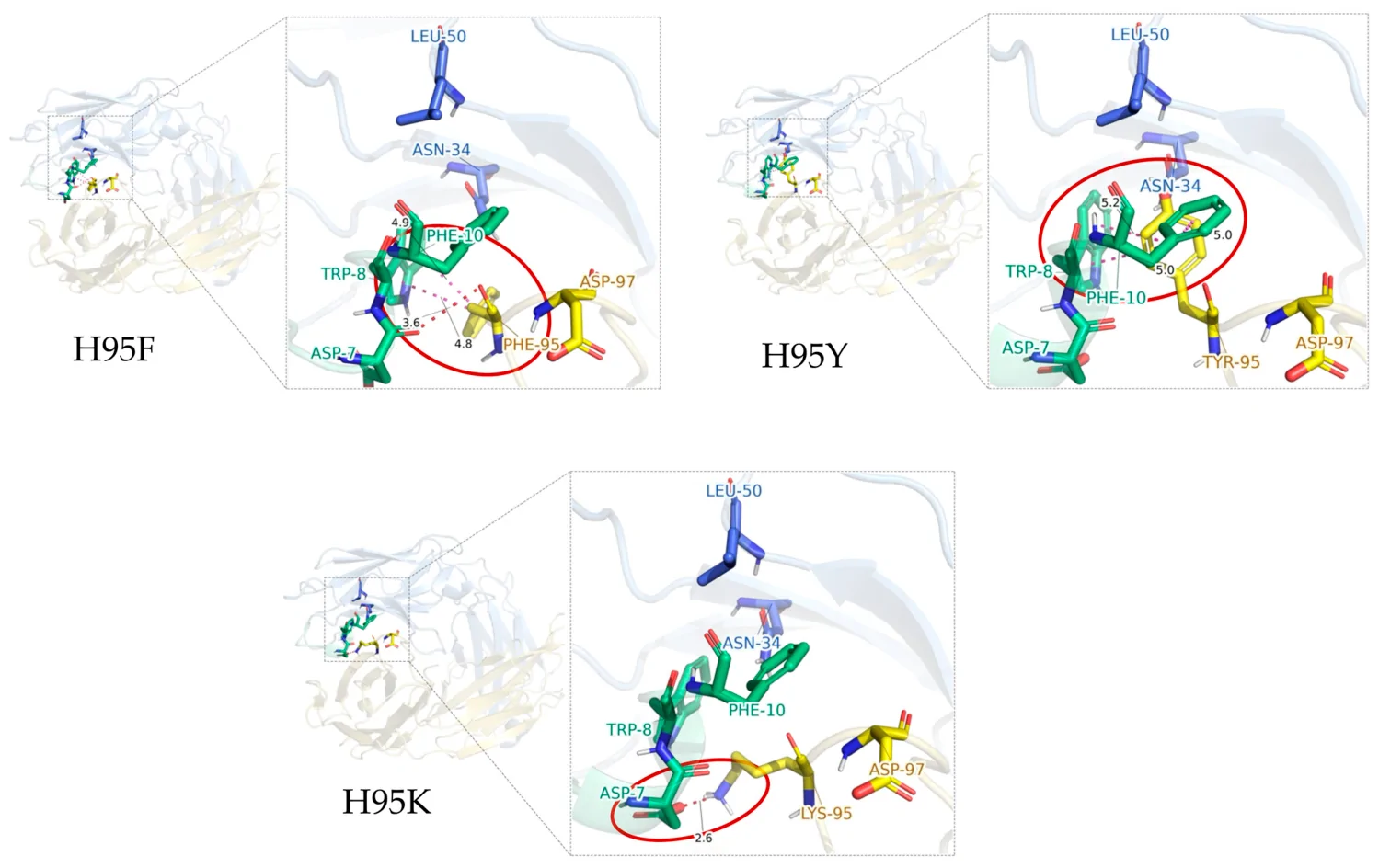
HADDOCK-refined structural comparison of H95 candidate mutations. Local structural views of H95F, H95Y and H95K are shown relative to the corresponding wild-type local interface environment. Each panel contains an overall view of the HzKR127–preS1 complex and an enlarged view of the local interface around residue H95. The red ellipses indicate the regions with prominent local contact changes. H95F and H95Y introduce aromatic side chains that may affect hydrophobic or aromatic contacts near the preS1 peptide, whereas H95K represents a charge-reversal substitution that may reshape local polar or electrostatic interactions. The heavy chain, light chain and preS1 peptide are shown in yellow, blue and green, respectively.

Residue H: E95 showed greater mechanistic heterogeneity (Figure 10). Wild-type Glu95 is an acidic, hydrophilic residue, whereas H: E95K, H: E95F and H: E95Y represent charge reversal and aromatic replacement. H: E95K may introduce a new polar contact or salt bridge with P: Asp7, thereby reshaping local electrostatic complementarity. H: E95F/Y may strengthen hydrophobic or aromatic contacts near P: Trp8 and P: Phe10. Thus, H: E95 may support optimization through either aromatic packing or local polar/electrostatic reconnection, making it suitable for extended validation.

**Figure 11:**
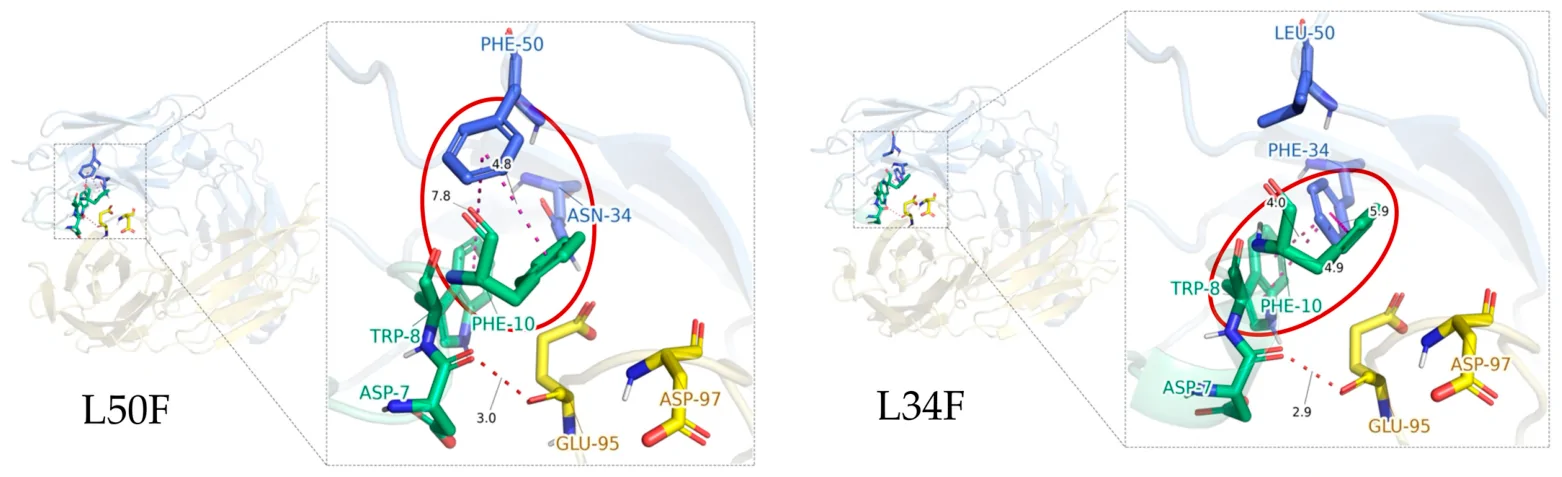
HADDOCK-refined structural comparison of light-chain candidate mutations. Local structural views of L50F and L34F are shown. Each panel contains an overall view of the HzKR127–preS1 complex and an enlarged view of the local interface around the corresponding light-chain residue. The red ellipses indicate the main regions of local contact changes after mutation. Introduction of phenylalanine at L50 or L34 may expand local aromatic or hydrophobic contacts near the preS1 peptide, suggesting that light-chain CDR residues may also contribute to interface stabilization. The heavy chain, light chain and preS1 peptide are shown in yellow, blue and green, respectively.

Light-chain candidates also suggested that LCDR regions may modulate interface stability (Figure 11). L: L50F and L: N34F introduce phenylalanine and expand the aro-matic/hydrophobic contact surface around P: Trp8 and P: Phe10, indicating that the light chain may contribute to antibody–peptide packing. L: L50R did not show an obvious new aromatic interaction, and its score improvement may instead reflect local polarity or broader contact network adjustment. Light-chain candidates therefore have potential but appear mechanistically less straightforward than H:D97 substitutions; they may be more appropriate as secondary or exploratory candidates.

**Figure 12:**
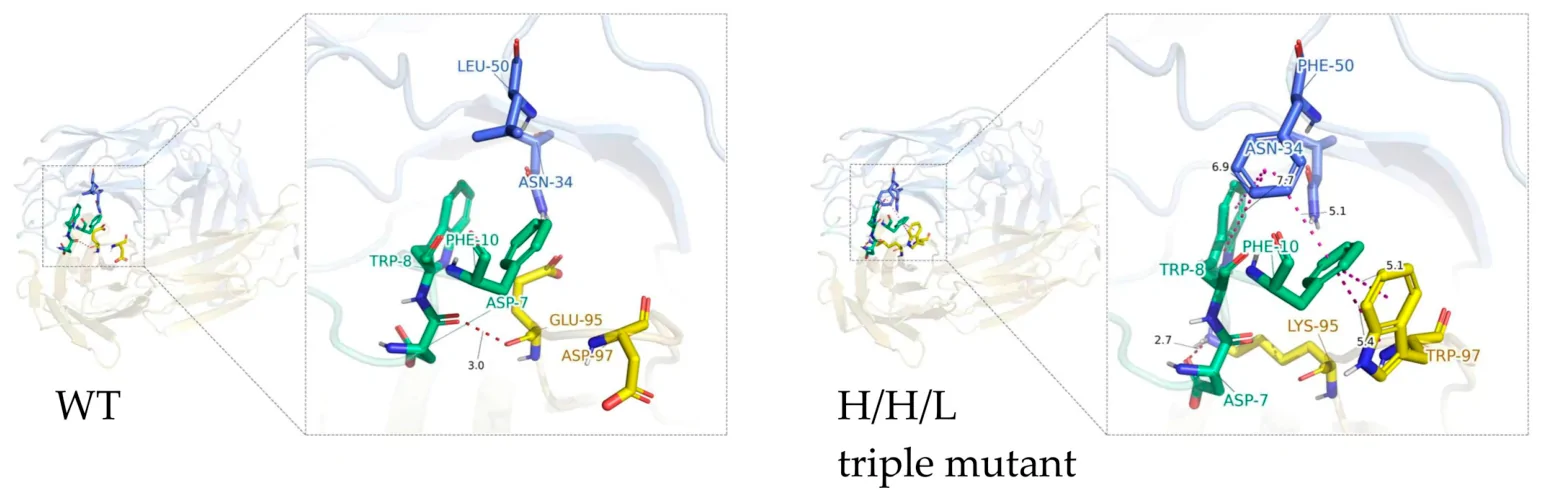
Structural comparison of wild-type and triple-mutant HzKR127–preS1 complexes. Panels display overall and magnified interface views. Combining the H95, H97, and L50 substitutions yielded a HADDOCK score of −185.15 (vs. −165.38 for wild-type). This model illustrates an in silico hypothesis for synergistic interface remodeling, not experimentally verified additive affinity. Scores indicate relative refinement stability rather than absolute binding free energies.

To evaluate potential cumulative effects, we modeled an H/H/L triple mutant combining the top-ranked substitutions at H95, H97, and L50 (Figure 12). Utilizing the same SF10 HADDOCK refinement protocol, the best-scoring structure of 100 generated models achieved a score of −185.15 kcal/mol, yielding a 19.77 kcal/mol gain over wild-type (−165.38 kcal/mol). Visual inspection suggests that these substitutions cooperatively remodel the interface through concurrent electrostatic rearrangement and enhanced aromatic/hydrophobic packing. While these coordinated changes suggest potential synergistic binding, they remain a structural hypothesis subject to subsequent experimental validation for true additive affinity.

Overall, high-scoring candidates clustered near the local epitope region defined by P:Phe10 and P:Trp8 of the preS1 peptide. Predicted gains primarily derived from enhanced aromatic and hydrophobic packing, supplemented by local polar rearrangements in select variants. Considering site-level consensus and structural interpretability, we prioritize H:D97W/F/Y for initial experimental validation, with H:E95K/F/Y and H:R50W serving as high-interest secondary candidates, and light-chain substitutions L:L50F, L:N34F and L:L50R as exploratory targets. Ultimately, downstream antibody optimization should transition from single-metric ranking reliance to multi-parameter evaluation integrating docking energy, local coordinate rearrangements, and physical structural hypotheses.

## 4. Discussion

Functional cure of chronic HBV infection remains a major challenge, with entry blockade representing a key antiviral strategy. The preS1 region mediates viral engagement of the NTCP receptor and contains critical neutralizing epitopes. The neutralizing antibody HzKR127 provides a valuable model to evaluate whether an antibody targeting a functionally constrained epitope can be optimized further. Because conventional virtual alanine scanning primarily detects disruptive effects rather than gain-of-function substitutions, and single-model predictions are often biased by unique structural assumptions, we combined saturation mutagenesis with multi-model consensus scoring to systematically map mutational responses, positional plasticity, and structural mechanisms across the HzKR127–preS1 interface [23].

The combination of saturation mutagenesis and multi-model consensus scoring provided a practical computational basis for candidate screening. Compared with single alanine scanning, the site-median score integrates the effects of multiple amino acid substitutions at the same residue and therefore more comprehensively reflects sensitivity to different side-chain chemistries. In the literature-based validation, alanine substitution and site-median scores yielded AUC values of 0.81 and 0.84, respectively, both clearly above the random baseline. Multi-model scoring reduced dependence on any single predictor and enabled model-support and model disagreement metrics to be used as indicators of prediction stability. However, because several methods share underlying structural assumptions, consensus integration cannot eliminate systematic biases or output-calibrated binding free energies; the scores should be interpreted as relative priorities rather than absolute affinity predictors. Nevertheless, this consensus framework provides a robust basis for prioritizing CDR candidates before structural reassessment and experimental validation.

A central feature of the HzKR127-preS1 interface is the coexistence of predicted antigen-side intolerance and antibody-side plasticity. Most substitutions on the preS1 peptide were predicted to impair HzKR127 recognition, suggesting an antibody-binding constraint rather than direct evidence of impaired NTCP engagement. Since binding-compatible mutations may still impair entry while disruptive ones remain viable, functional assays are required to directly link this profile to viral fitness. In contrast, the HzKR127 CDRs retained localized optimization signals. Regions around H95, H97, L28 and L34 present packing-deficient or chemically reconfigurable microenvironments. While aromatic and hydrophobic enrichment near preS1 Trp8/Phe10 may reflect genuine interface complementarity—consistent with native antibody paratopes [24], it might also stem from shared predictor biases toward packing and desolvation; hence, aromatic enrichment alone cannot guarantee improved affinity. Instead, refining specific CDR microenvironments via polar or charged substitutions to optimize electrostatic and hydrogen bond networks offers a more viable engineering path than uniform interface strengthening.

Residue-level partitioning confirms that binding hot spots are not automatically optimal engineering sites. While some act as rigid anchors stabilizing the complex, other hot spots or warm spots may lie in reconfigurable regions and tolerate specific substitutions that improve shape complementarity, hydrophobic packing or polar matching [25,26,27]. Candidate prioritization should therefore integrate three criteria: baseline interface contribution, predicted substitution gain, and inter-model consensus support. Accordingly, the published alanine-scanning benchmark validates the identification of key binding residues but does not verify the gain-of-function ranking of non-alanine substitutions.

The prioritization of H97Y and H97W is consistent with this mechanistic framework. The recurrence of H97 in both previous affinity maturation and the present prioritization suggests that this HCDR3 position is a design-sensitive node, although the optimal substitution type requires direct experimental validation. H97 lies within the heavy-chain CDR contact region and is sensitive to aromaticity and side-chain volume. Tyrosine may combine aromatic contact with polar compatibility, whereas tryptophan may strengthen aromatic stacking and hydrophobic packing but may also introduce steric risk. Importantly, HADDOCK scores represent qualitative indicators of structural plausibility rather than calibrated measures of experimental binding free energy. This limiting caveat is highly relevant for the highly flexible HCDR3 loop, which can undergo conformational adjustments during antigen recognition. Substitutions at H:D97 may alter local side-chain packing but can also induce backbone shifts or induced-fit effects not fully captured by static, localized refinement. Therefore, these score improvements support structural interpretation and candidate prioritization hypotheses, rather than experimentally confirmed affinity enhancement. Surface plasmon resonance, biolayer interferometry or related kinetic assays will be required to determine their true binding effects. Expression level, thermal stability and nonspecific binding should also be assessed to evaluate engineering feasibility [23,26,28].

Considering that our prioritized candidates are limited to computational predictions, full validation of these mutations requires downstream kinetics (e.g., SPR, BLI, ELISA) and in vitro viral neutralization assays, alongside critical developability filters (expression titers, monomeric content via SEC-HPLC, thermal stability via DSF, and solubility) to ensure folding is not compromised. Notably, replacing polar or charged residues with bulky aromatics (Trp, Tyr, Phe) is a well-established affinity-maturation strategy. Chen et al. [29] demonstrated in anti-VEGF CDRs that polar-to-aromatic substitutions optimized shape complementarity and packing, yielding up to 100-fold affinity improvements without compromising expressibility. Similarly, Lippow et al. [30] designed and experimentally verified that introducing similar aromatic substitutions achieved multi-fold binding gains with favorable biophysical properties. These precedents provide a strong biophysical foundation for our prioritized candidates (such as H:D97W, H:D97Y, and L:S50W), indicating they are grounded in translatable, molecularly validated principles.

In summary, using the neutralizing antibody HzKR127 as a model, this study demonstrated that antibodies targeting functionally constrained preS1 epitope retains additional optimization potential. Within this system, integrating saturation-mutagenesis profiles, multi-model consensus scoring and structural reassessment provided a practical pipeline for evaluating mutation effects, model stability, and structural plausibility prior to experimental testing. Whether this workflow is fully transferable to distinct antibody–antigen systems remains to be independently validated.

## 5. Conclusions

We established a computational candidate-prioritization workflow for the HzKR127–preS1 interface by combining single-site saturation mutagenesis, multi-model consensus scoring and HADDOCK-based structural reassessment. Benchmarking against published alanine scanning data supported the ability of the consensus score to recover literature-annotated binding-contribution residues. The interface showed asymmetric predicted mutational responses: the preS1 peptide was broadly intolerant to substitution, whereas antibody CDRs retained localized designable regions. The prioritized variants should be regarded as experimentally testable candidates rather than validated affinity-enhancing mutations, and their effects on binding affinity, neutralization activity, expression, stability and developability require future experimental validation. Overall, this study provides a computational basis for prioritizing experimentally testable HzKR127 variants. Extension of the workflow to other antibody–antigen systems will require additional validation.

## Figures and Tables

**Table 1:** HADDOCK refinement scores, energy decomposition and local interaction changes for the top 10 candidate mutations.

Rank	Mutation	HADDOCK Score	Score Gain	vdW Gain	Electrostatics Gain	Desolvation Gain	BSA Δ	H-Bond Δ	Salt Bridge Δ	Hydrophobic Δ	Aromatic Δ
1	L:L50F	−173.808	8.43	0.29	28.19	2.51	10.71	4	0	7	1
2	H:D97W	−173.333	7.96	1.45	3.58	5.59	42.10	0	0	1	1
3	H:D97F	−172.259	6.88	4.05	−3.41	3.52	17.37	1	0	5	1
4	H:E95K	−171.122	5.74	−2.69	24.42	3.58	9.27	0	1	10	0
5	H:D97Y	−170.955	5.58	2.70	1.79	2.53	99.32	2	0	10	1
6	H:E95F	−169.635	4.25	1.24	3.88	2.24	1.74	1	0	9	0
7	L:N34F	−169.405	4.03	−0.05	−7.36	6.17	21.28	1	0	19	0
8	L:L50R	−168.911	3.53	0.83	2.84	2.13	45.82	−1	0	1	0
9	H:R50W	−168.882	3.50	8.81	−48.76	6.46	34.53	−1	−1	17	1
10	H:E95Y	−168.869	3.49	0.01	−8.43	5.17	57.53	1	0	12	0

Note: Mutations are ranked by HADDOCK score from low to high. Score gain indicates the improvement in HADDOCK score relative to the wild-type complex. BSA Δ indicates the change in buried surface area. H-bond Δ, salt bridge Δ, hydrophobic Δ and aromatic Δ indicate changes in the numbers of hydrogen bonds, salt bridges, hydrophobic contacts and aromatic interactions after mutation. Negative values indicate fewer interactions after mutation.

## Data Availability

The structural template used in this study is publicly available from the Protein Data Bank under accession code 2EH8. The published alanine scanning data analyzed in this study are available from the cited literature. Additional processed data generated during the current study are available from the corresponding author upon reasonable request.

## Supplementary Materials

The following supporting information can be downloaded at https://www.mdpi.com/article/10.3390/cimb48080791/s1.

## Abbreviations

The following abbreviations are used in this manuscript:

HBV	Hepatitis B virus
NTCP	Sodium taurocholate cotransporting polypeptide
HzKR127	Humanized KR127
Fab	Fragment antigen-binding
PDB	Protein Data Bank
CDR	Complementarity-determining region
HCDR	Heavy-chain complementarity-determining region
LCDR	Light-chain complementarity-determining region
AUC	Area under the curve
ROC	Receiver operating characteristic
BSA	Buried surface area
vdW	van der Waals
WT	Wild type
