# Supplementary material for: A Multi-Model Strategy for Optimizing Hepatitis B Virus preS1 Epitope Recognition by the Antibody HzKR127
Source: Curr Issues Mol Biol. 2026 Aug 3;48(8):791. doi: 10.3390/cimb48080791 (PMC13511535; doi:10.3390/cimb48080791)
Supplement: Supplementary file 1 [file cimb-48-00791-s001.zip › Supplemental Figures.pdf]

Supplemental Figures

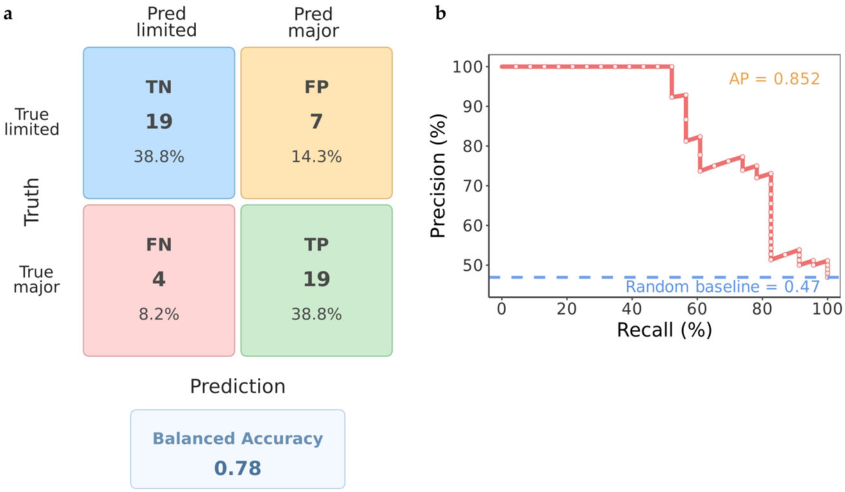

Supplementary Figure S1. Supplementary validation of the multi-model consensus scoring and candidate-ranking strategy. (a) Confusion matrix at the Youden index–optimized threshold for classifying major-affinity-loss and limited-affinity-loss sites. (b) Precision–recall curve for identifying experimentally defined major-affinity-loss sites using the consensus score. The average precision was 0.852.

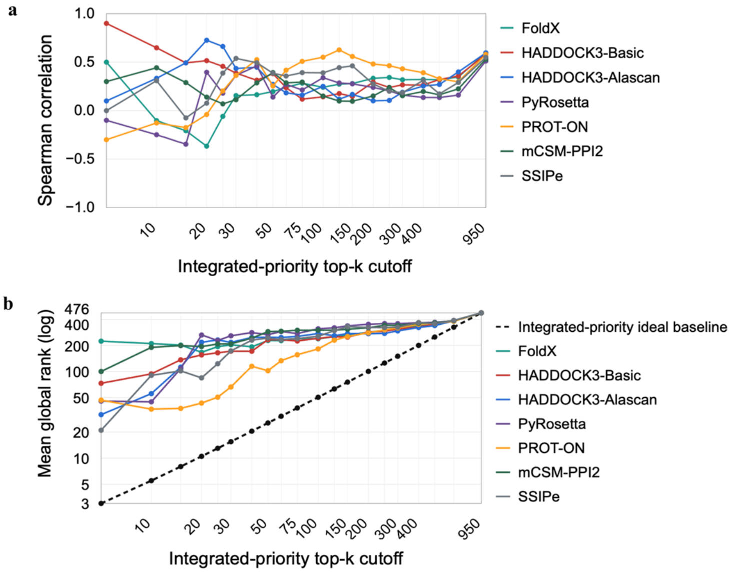

Supplementary Figure S2. Model-consistency analysis of the integrated-priority ranking. (a) Spearman correlation between individual model rankings and the integrated-priority ranking across different top-k cutoffs. (b) Mean global rank of candidates selected by the integrated-priority top-k cutoff in each individual model. The dashed line indicates the ideal integrated-priority baseline. FoldX, HADDOCK3-Basic, HADDOCK3-Alascan, PyRosetta, PROT-ON, mCSM-PPI2 and SSIPe were included in the comparison.
